# The Effects of Cognitive Behavioral Therapy for Insomnia on Physical Activity Before and After Time in Bed Among Shift Workers

**DOI:** 10.3390/jcm14093206

**Published:** 2025-05-06

**Authors:** Marcin Sochal, Bernd Feige, Kai Spiegelhalder, Johanna Ell

**Affiliations:** 1 Department of Sleep Medicine and Metabolic Disorders, Medical University of Lodz, 90-419 Lodz, Poland; 2 Department of Psychiatry and Psychotherapy, Medical Center—University of Freiburg, Faculty of Medicine, University of Freiburg, 79106 Freiburg, Germany; bernd.feige@uniklinik-freiburg.de (B.F.); kai.spiegelhalder@uniklinik-freiburg.de (K.S.); johanna.ell@uniklinik-freiburg.de (J.E.)

**Keywords:** insomnia, physical activity, CBT-I

## Abstract

**Background:** Sleep and physical activity (PA) are bidirectionally related, with PA having a positive effect on sleep, and sleep quality influencing PA the following day. However, little is known about the effects of clinical interventions for sleep disorders on PA. Therefore, the aim of this secondary analysis is to evaluate the impact of cognitive behavioral therapy for insomnia (CBT-I), the first-line treatment for insomnia, on PA. **Methods:** Thirty-eight nurses with shift work disorder and insomnia were randomly assigned to either CBT-I or a waitlist control group. PA was measured for one week before (T0) and after the intervention/waiting period (T1) using actigraphy and sleep diary items. The impact of CBT-I on the PA parameters was analyzed using linear mixed models. In addition, correlations of pre-to-post-treatment changes in PA and pre-to-post-treatment changes in the clinical outcomes (insomnia severity, sleep efficiency, depression) were explored in the CBT-I group. **Results:** CBT-I increased actigraphy-derived PA during the two hours (β = 26.17, SE = 9.41, *p* = 0.009) and one hour (β = 13.24, SE = 4.57, *p* = 0.006) after time in bed, and resulted in a higher percentage of self-reported days with PA (β = 19.11, SE = 9.36, *p* = 0.049) compared to the waitlist control group. No significant correlations were found between the changes in PA and clinical outcomes, except for a moderate positive correlation between changes in self-reported sleep efficiency and changes in PA one hour before time in bed (r = 0.56, *p* = 0.013). **Conclusions:** This is the first study to investigate the impact of CBT-I on PA, providing preliminary evidence of the potential positive effects. Further studies with larger sample sizes and randomized controlled designs with continuous PA monitoring are needed to confirm these preliminary results.

## 1. Introduction

Sleep and physical activity (PA) are essential features of human life, and there is probably a bidirectional association between them. With respect to the effects of PA on sleep, meta-analytic evidence shows that regular exercise improves both subjective and objective sleep parameters in healthy individuals [1]. Importantly, contrary to the existing sleep hygiene recommendations, even high-intensity exercise in the evening does not seem to have negative effects on subsequent sleep [2]. In addition, PA has been used in the clinical context with reasonable efficacy to improve sleep onset and sleep maintenance difficulties in patients with insomnia [3].

Vice versa, the results for the effects of sleep on PA are less clear. In healthy individuals, high sleep efficiency and high sleep quality are associated with increased PA the following day [4]. Additionally, PA during the day was associated with shorter total sleep time the following night [4]. However, the corresponding study results are somewhat inconsistent, and little is known about the effects of clinical interventions for sleep disorders on PA. A meta-analysis conducted in a group of university students showed a weak negative association between moderate to vigorous physical activity and the sleep duration [5]. However, the results of the corresponding studies are somewhat inconsistent, and little is known about the effects of clinical interventions for sleep disorders on PA. The first-line treatment for insomnia disorder is cognitive behavioral therapy for insomnia (CBT-I) [6]. In light of the robust effects of CBT-I on sleep onset and sleep maintenance difficulties [7], it is conceivable that CBT-I also has a positive effect on PA. However, to our knowledge, the potential effects of CBT-I on PA have not been systematically investigated so far.

Therefore, the aim of the current study was to evaluate the impact of CBT-I on PA. For this purpose, a secondary analysis was conducted using actigraphy-derived and self-report PA data from a randomized controlled trial that investigated the efficacy of CBT-I in shift workers [8].

## 2. Methods

This was a secondary analysis of a randomized controlled parallel-group trial comparing digitally delivered guided CBT-I with a waitlist control condition in nurses suffering from shift work disorder with insomnia. Shift work disorder is defined as insomnia and/or excessive sleepiness associated with a work schedule that persists for a minimum of three months [9]. The trial was carried out in accordance with the Declaration of Helsinki and registered in the German Clinical Trials Register (https://www.drks.de/drks_web/; accessed on 18 October 2021 ID: DRKS00026770). The study protocol was approved by the local ethics committee (Leuphana University of Lüneburg, EB-Antrag_202007-12-Lehr_ONSEPS). Written informed consent was obtained from all the participants prior to their inclusion in the study, including the use of collected data for secondary analyses over a 10-year period.

### 2.1. Participants

Nurses were informed about the study through leaflets, emails, and the social media accounts of hospitals within Southern Germany. Subsequently, the eligibility for study participation was evaluated during a telephone-based screening interview conducted by a clinical psychologist specialized in sleep medicine (author JE). Next, the participants were randomly assigned to the intervention group (the guided digital CBT-I program SleepCare) or the waitlist control group using simple randomization performed by an independent individual not otherwise involved in the study, thereby ensuring allocation concealment. Due to the nature of the study design, blinding was not feasible. The recruitment for the study took place between October 2021 and November 2022 in Germany. Participants were eligible for inclusion when they met the following criteria: (1) age between 18 and 65 years; (2) employment as a nurse; (3) rotating shift work during the entire study participation (working at least in a half-time position) with at least 10% of the shifts being night shifts (defined as a minimum of 3 h of working time between 22:00 h and 06:00 h); (4) a diagnosis of shift work disorder with insomnia (with or without daytime sleepiness); (5) internet access; and (6) fluency in the German language. The exclusion criteria were: (1) any other comorbid psychiatric or sleep disorder; (2) any severe or unstable somatic disease that influences sleep; (3) the intake of medication affecting sleep in the 2 weeks before or during the study participation; (4) acute suicidality; (5) previous treatment with CBT-I; (6) current psychotherapeutic treatment or being on a waitlist for psychotherapy; and (7) current participation in another health training program to improve sleep. For the original study, a sample size was calculated for *n* = 46; for a detailed explanation of the calculation, see Ell et al., 2024 [8].

### 2.2. Intervention and Control Condition

Intervention and control condition: After inclusion, the study participants were randomly assigned to the intervention group (the guided digital CBT-I program SleepCare) or the waitlist control group through simple randomization by a person not involved in the project. SleepCare is a digital CBT-I program delivered through an interactive website, designed for nurses suffering from shift work disorder with insomnia. The program comprises six self-paced modules, including text resources, explanatory videos, interactive exercises, and audio files. A detailed description of the SleepCare modules was provided in our previous report [8].

### 2.3. Outcomes

The pre-treatment assessment (T0) included the completion of a one-week sleep diary [10] and, simultaneously, a one-week activity monitoring using actigraphy, a wearable device, which is worn on the wrist and records movement to estimate the sleep and activity patterns (Movisens Light 3 Sensor; Movisens GmbH, Karlsruhe, Germany). Basic demographic data (e.g., age, gender, BMI) were collected, and the participants were asked to complete several questionnaires, including the Insomnia Severity Index (ISI) [11] and the Beck Depression Inventory-II (BDI-II) [12]. Other questionnaires completed by the participants were not the focus of this secondary analysis and are described in detail in our previous report [8]. The post-treatment/post-waitlist assessment (T1) was conducted again according to the T0 protocol (excluding demographic data) as soon as possible after the completion of the treatment/the 8-week waitlist condition.

### 2.4. Statistical Analysis

Sleep diaries were analyzed to obtain data on sleep efficiency (SE). The data were manually checked for plausibility, and unrealistic entries (e.g., TST of 1400 min, SOL of 10,000 min) were removed. In the sleep diaries, patients also answered daily questions on PA: how much time they spent in PA on a given day, whether they were active that day, and whether they were active in the two hours before going to bed. Based on these questions, continuous variables—the average daily PA time and the average number of days during which the patient was active (or was active in the two hours before going to bed)—were calculated.

The actigraphy data were preprocessed using the pyActigraphy package [13] in Python 3 (https://www.python.org/ accessed on 1 May 2025). The data were manually validated, and unrealistic data were removed, e.g., a TST > 1000 min. Due to reasons relating to labor law and hygiene, the participants did not wear the actigraphs during working hours. Because of this, the following actigraphy-derived PA parameters (continuous variables) were calculated for each day: average PA as well as minutes of >10 mg PA (see [14]) one and two hours before and one and two hours after time in bed (TIB). In cases in which the periods of one or two hours before/after TIB overlapped with the time when the actigraph was not worn, such data were removed from the analysis. Participants with data available for less than 4 out of 7 days were excluded from the analyses. For two participants, actigraphy data were not obtained at T1 and imputed by using the last-observation-carried-forward method.

Pre-to-post-treatment effects were examined using linear mixed-models with the independent variables “group” (CBT-I vs. waitlist control) and “time” (T1 versus T0). These models included the “group × time” interaction as a random effect. In addition, the correlations between the pre-to-post-treatment changes in the PA parameters and pre-to-post-treatment changes in the clinical outcomes (ISI, BDI, sleep diary-derived SE, actigraphy-derived SE) were explored in the intervention group using the Pearson correlation test. The data analysis was conducted using R version 4.4.2 (http://www.R-project.org/ accessed on 1 May 2025) and Statistica 13.1 PL (StatSoft, Tulsa, OK, USA). A *p*-value below 0.05 was considered statistically significant.

## 3. Results

Of the 46 participants from the parent trial, 8 were excluded from the current analysis because of substantial missing actigraphy data. Thus, a total of 38 participants were included in the analysis of which 19 participants underwent the intervention. Both groups consisted of 15 women and 4 men. The mean age at T0 was 40.1 ± 12.1 years in the CBT-I group and 42.2 ± 11.9 years in the waitlist control group.

In the analyses of the actigraphic PA parameters, there were significant group × time interaction effects for minutes of >10 mg PA during the two hours after TIB (β = 26.17, SE = 9.41, *p* = 0.009) and for minutes of >10 mg PA during the one hour after TIB (β = 13.24, SE = 4.57, *p* = 0.006), highlighting an increase of >10 mg PA after TIB in the CBT-I group and a decrease of >10 mg PA in the waitlist control group. No significant group × time interaction effects were observed for the other actigraphy-derived PA parameters (see Table 1). In the analyses of sleep diary-derived PA parameters, there was a significant group × time interaction effect for the percentage of days with PA (β = 19.11, SE = 9.36, *p* = 0.049) highlighting an increase of days with PA in the CBT-I group and a decrease of days with PA in the waitlist control group. No significant group × time interaction effects were observed for other sleep diary-derived PA parameters (see Table 1).

The correlational analyses between the pre-to-post-treatment changes in PA and pre-to-post-treatment changes in clinical outcomes in the intervention group produced mostly non-significant results (see Table 2). The only exception was the analysis of pre-to-post-treatment changes in PA one hour before TIB in relation to the pre-to-post-treatment changes in sleep diary-derived SE, which revealed a significant positive correlation (r = 0.56, *p* = 0.013).

## 4. Discussion

This study investigated the impact of CBT-I on PA. Using actigraphy-derived and self-report PA data from a randomized controlled trial with shift workers, we analyzed the pre-to-post-treatment changes in a CBT-I group compared to a waitlist control group. Overall, the findings indicate that CBT-I increases PA in comparison with a waitlist control condition. However, the pre-to-post-treatment changes in PA parameters showed no consistent associations with the pre-to-post-treatment changes in clinical outcomes within the intervention group.

More precisely, CBT-I increased the actigraphy-measured PA one hour and two hours after TIB and the percentage of self-reported days with PA compared to the waitlist control group. Several factors may contribute to these findings. First, increased activity levels might result from the positive effects of CBT-I on sleep, leading the participants to feel more rested, which might have increased their motivation and energy to engage in PA [7,14,15]. Second, during the sleep restriction therapy, the participants were advised to engage in light activity rather than lying down when feeling fatigued, helping them stay awake and maintain sleep pressure [16,17]. Sleep restriction therapy also leads to a reduced time in bed, which simply increases the available time for PA. Finally, sleep hygiene recommendations generally emphasize the positive effects of regular PA on sleep [18,19]. Of note, the less pronounced difference between the groups in the evening before TIB may also be related to a sleep hygiene recommendation, i.e., to avoid intense exercise before sleep.

With respect to the associations between the pre-to-post-treatment changes in actigraphy- and sleep diary-derived PA parameters and the pre-to-post-treatment changes in the clinical outcomes within the intervention group, no consistent pattern of findings was observed. A moderate positive correlation was found between the changes in sleep diary-derived sleep efficiency and changes in PA one hour before TIB. However, no other significant correlations between any of the investigated variables were identified. These results may be attributed to the sample size of these analyses which was very small for investigating between-subject correlations (n = 19) reducing the statistical power. Another reason for the absence of an association may be that the changes in PA are influenced by other factors than direct improvements in sleep (e.g., psychoeducation about healthy lifestyle and PA effects on sleep that may directly stimulate increased PA). Nevertheless, the observed correlation may provide an initial indication that increased activity and higher sleep efficiency could be related, which is consistent with the other findings in our study.

The preliminary evidence indicates that CBT-I might not only improve sleep but could also have a positive impact on the PA levels, possibly mediated by improvements in energy, motivation, and daytime functioning [7,20,21]. Given the well-established benefits of PA on various health outcomes, including cardiovascular health, mental well-being, and metabolic regulation (e.g., [22,23]), these effects of CBT-I on PA could have far-reaching implications for overall health. Since the results of this study indicate that CBT-I increases PA among shift workers, it is plausible to hypothesize that similar effects might occur in non-shift workers suffering from insomnia disorder. However, given that non-shift workers already tend to be more physically active than shift workers [24], the impact of CBT-I might be smaller (due to a ceiling effect) or even non-significant. Alternatively, CBT-I may not increase the overall physical activity but rather improve aspects such as the regularity or structure of activity patterns. Future research is needed to systematically investigate these potential effects.

To the best of our knowledge, this is the first study to investigate the effects of CBT-I on the intensity of PA. In doing so, we drew on data from a randomized trial and assessed A using both objective and subjective measures. However, it is important to acknowledge certain limitations of this study. First, as a secondary and exploratory analysis, the causal inferences are limited, and the potential confounders may not be fully accounted for. Second, the reduced sample size, partly due to missing actigraphy data, may have introduced a selection bias and reduced statistical power. Third, the study population consisted exclusively of shift workers. While this focus allows for detailed insights into this particular subgroup, it also limits the generalizability of our findings to the broader population. Last, actigraphy devices had to be removed during working hours by the participants, potentially leading to data loss that could have revealed stronger between-group differences.

## 5. Conclusions

In summary, CBT-I can increase the level of PA measured by using objective methods (actigraphy) and subjective methods (sleep diaries). In light of these promising yet preliminary findings, larger studies with randomized controlled designs are needed to validate our results, incorporating the continuous measurement of PA and assessing its impact on general well-being and overall health.

## Figures and Tables

**Table 1:** Effects of cognitive behavioral therapy for insomnia on actigraphy- and sleep diary-derived PA parameters.

			CBT-I Group (n = 19)			Waitlist Control Group (n = 19)			Group × Time Interaction			Effect Size
			T0	T1		T0	T1		ß	SE	*p*	Cohens’ d
Actigraphic parameters	Before TIB	PA two hours before TIB	7.6 (4.0)	10.5 (7.1)		11.6 (9.0)	9.5 (6.5)		4.95	2.81	0.087	0.57
		Minutes of >10 mg PA during the two hours before TIB	17.3 (9.4)	22.5 (13.5)		25.8 (18.3)	21.0 (13.2)		10.01	5.67	0.086	0.57
		PA one hour before TIB	4.5 (3.1)	7.7 (8.6)		6.2 (5.5)	5.6 (3.2)		3.90	2.52	0.131	0.50
		Minutes of >10 mg PA during the one hour before TIB	5.4 (4.3)	7.0 (6.5)		8.0 (7.3)	6.2 (5.4)		3.38	2.38	0.164	0.46
	after TIB	PA two hours after TIB	36.0 (33.0)	41.8 (31.2)		52.0 (23.1)	45.4 (28.8)		12.30	7.16	0.095	0.56
		Minutes of >10 mg PA during the two hours after TIB	55.4 (28.8)	63.7 (26.3)		74.5 (26.7)	56.6 (31.8)		**26.17**	**9.41**	**0.009**	**0.90**
		PA one hour after TIB	36.3 (34.2)	46.9 (36.0)		57.0 (27.0)	52.5 (42.3)		15.16	10.02	0.139	0.49
		Minutes of >10 mg PA during the one hour after TIB	29.6 (12.7)	34.4 (13.1)		40.3 (12.1)	31.9 (15.3)		**13.24**	**4.57**	**0.006**	**0.94**
Sleep diary parameters		Average time of PA (minutes per day)	18.3 (20.4)	27.0 (37.4)		25.2 (21.9)	28.5 (34.5)		5.32	9.78	0.590	0.18
		% of days with PA	22.5 (19.0)	31.7 (36.9)		39.5 (28.9)	29.6 (27.0)		**19.11**	**9.36**	**0.049**	**0.66**
		% of days with PA two hours before TIB	30.4 (34.1)	27.1 (33.0)		20.9 (30.7)	30.1 (38.1)		−12.47	10.29	0.234	-0.39

Bold—statistically significant. Data are presented as mean (SD). CBT-I—cognitive behavioral therapy for insomnia, PA—physical activity, T0—pre-treatment assessment, T1—post-treatment/post-waitlist assessment, TIB—time in bed.

**Table 2:** Correlations between changes in actigraphy- and sleep diary-derived PA parameters from pre-to-post-treatment and changes in clinical outcomes from pre-to-post-treatment in the CBT-I group (n = 19).

			ISI Changes			BDI Changes			SE Changes (Diary)			SE Changes (Act)	
			r	*p*		r	*p*		r	*p*		r	*p*
Changes in actigraphic parameters	Before TIB	PA two hours before TIB	0.05	0.830		−0.17	0.487		0.15	0.545		−0.12	0.626
		Minutes of >10 mg PA during the two hours before TIB	0.09	0.726		−0.01	0.965		0.07	0.771		−0.29	0.236
		PA one hour before TIB	−0.05	0.855		−0.27	0.257		**0.56**	**0.013**		−0.01	0.976
		Minutes of >10 mg PA during the one hour before TIB	−0.04	0.875		−0.00	0.988		0.26	0.276		−0.36	0.130
	after TIB	PA two hours after TIB	0.36	0.126		−0.09	0.729		−0.24	0.317		0.03	0.919
		Minutes of >10 mg PA during the two hours after TIB	0.04	0.882		−0.14	0.574		0.08	0.757		−0.17	0.481
		PA one hour after TIB	0.26	0.288		−0.18	0.462		−0.08	0.732		−0.05	0.826
		Minutes of >10 mg PA during the one hour after TIB	0.20	0.402		−0.29	0.229		0.19	0.442		−0.10	0.688
Changes in sleep diary parameters		Average time of PA (minutes per day)	0.06	0.806		0.24	0.313		−0.21	0.382		0.28	0.251
		% of days with PA	0.34	0.161		0.44	0.061		−0.45	0.055		0.23	0.342
		% of days with PA two hours before TIB	0.20	0.409		−0.14	0.574		0.16	0.524		−0.21	0.379

Bold—statistically significant. Abbreviations: act—actigraphy, BDI—Beck Depression Inventory-II, ISI—Insomnia Severity Index, PA—physical activity, SE—sleep efficiency, TIB—time in bed.

## Data Availability

The data presented in this study are available on request from the corresponding author.

## Abbreviations

act	actigraphy
BDI-II	Beck Depression Inventory-II
BMI	Body Mass Index
CBT-I	cognitive behavioral therapy for insomnia
ISI	Insomnia Severity Index
SD	standard deviation
PA	physical activity
SE	sleep efficiency
SOL	sleep onset latency
T0	pre-treatment assessment
T1	post-treatment/post-waitlist assessment
TIB	time in bed
TST	total sleep time
